# Mr. Waiblinger, on the Effects of Galvanism

**Published:** 1806-02

**Authors:** J. Waiblinger

**Affiliations:** Fulneck, near Leeds


### *To the Editors of the Medical and Phyſical Journal.*

Gentlemen,

I Was very much pleased to see in the Journal for this month, Mr. Whitlam's Case of Epilepsy cured by the Galvanic process, for which he has my best thanks. Having for above a year back paid a good deal of attention to that discovery, and having constructed a very powerful Galvanic trough, I have tried its effects in a great variety of cases, and in many with very satisfactory results. If you think the following cases worth your insertion they are at your service. I beg to make my acknowledgements to Mr. C. Wilkinson for his excellent work upon the subject, from which I have received great assistance, both in the construction and application of my machine. So powerful an agent as Galvanism in my opinion is, deserves more attention than has hitherto been paid to it, in its application to the healing art, and therefore I should be very happy if many more would contribute the results of their experience through the medium of your excellent Journal. At present we are as it were only feeling our way, for its proper application. I am fully convinced by experience of its utility in many cases, and am also convinced it may do do injury in others; but what article of the Materia Medica, of the powerful class, will not do the same if abused?

In chronic rheumatisms in elderly people of an old standing, great relief has been experienced. Indolent tumors of various descriptions have been in some cases totally removed, and in many others much lessened by its application, which encourages a perseverance in its use. The two following cases of *Gutta Serena,* I hope, will however not be deemed uninteresting; as far as I know, none have been published. In cases of deafness I have had but little experience, though from analogy, I should suppose it would be useful in many cases. I tried it with one old gentleman, but as he found no benefit after trying it two or three times, he came no more; but I do not consider this a fair trial.

Case I.—Joseph Haley, ætat. 43, of Hunsworth, by trade a farmer, applied to me about two years ago, for a dimness of sight in both eyes; the left was the worst, the pupil of that eye was more dilated than the right, and the expansion of the retina at the bottom of the eye, had a dull whitish appearance; he could see a little with the right eye, but on shutting it he could scarce tell day from night; he complained of a dull pain in his head, and slight pains on moving his eyes; to use his own expression, *quite at the bottom of his eyes.* His health was tolerably good, his bowels however rather confined; the nasal discharge was unusually trifling, and he said had been so, as far as he could recollect, for several years. He imagined that his eyes had received some injury from spreading lime upon his grounds. I gave him a brisk purgative, and the following errhine snuff:

℞. Hydrargyri vitriolati ℈ß. pulv. hellebor. alb. ℈j. pulv glycyrhizæ ℈jß. M. cujus parv. (usquè gr. vj. vel viij.) in nares attrahantur quotidiè, vel bis in die si opus ferat.

I saw him again in about a week; he said he found his head much relieved, and his right eye a great deal better, the left not worse but nearly the same. I saw him no more till August last; his wife came over to me about the middle of the month, and informed me, that he had totally lost the sight of his right eye, and wished me to prescribe something for him. I told her that I rather wished to see him first; and as I had to galvanise some other patients the next morning, I directed him to come at a certain hour. On his arrival, led by his wife, he seemed very low

 spirited.

spirited. The pupil of the right eye very large and insensible to the light of a candle, but he could still with the left distinguish day-light. I placed him under the influence of the Galvanic fluid for about twenty minutes; and on his rising from the chair, he told me he thought he saw me move on the floor. I directed him to attend again the next day but one; he then informed me that he certainly saw more light, and he had a sensation of flashes of fire frequently before his eyes. He continued attending every other day for some time, and every time shewed evident improvement, the iris contracting more considerably every day. After the fifth time he was able to walk over here alone, though with some difficulty; the sixth time of his attendance, on his arrival I happened to be operating upon a gentleman who had had a violent attack of hemiplegia, who came to my house in a gig; the day being warm the horse was hung to an iron ring near my door, and Haley sat near it with the door open; I was agreeably surprised to hear him call out, Mr. B. your servant must attend to the horse, or the reins will be broke; he has got loose, and is treading them under his feet. I immediately went to the door, and found the man was in the right, to my no small satisfaction. He attended in all twelve times; the last time he said he could see as well as he ever did, but as he was come he would have another bout. I heard from him lately and he continues very well. I gave him no medicines whatever, wishing to give Galvanism a fair trial. I do not call in question that it may be occasionally assisted, but as from the first he seemed to receive so much benefit, I did not make use of any collateral aid.

Case II.—John Bradshaw, ætat. 45, of Wibsey Low Moore, husbandman, had the misfortune to lose his left eye by the small-pox, when a boy; he applied to me in June last, as his other eye was nearly quite dark. On examining his eye, I found the pupil very large and nearly insensible, the iris itself seemed to be diseased; whether it was the complaint understood by the old term *Mydriasis*, I did not know, but I was convinced there existed an almost total paralysis of the optic nerve, and therefore recommended Galvanism, which was used every other day for near five weeks, and the result was equally satisfactory with the other case; the iris also (whether from increased absorption or what cause else, I do not wish to enter into) assumed its proper appearance, and the patient sees now very very well. I could give many more cases, but let these suffice for the present; my reason for giving the later in point of date first, was, because I think it the more striking.

I shall just observe, that to so delicate an organ it is most proper to begin very gently, and increase the power as the patient can bear it. My trough contains about 1280 square inches of metallic surface; at first I did not use above four or five pair of plates; and drew the shocks principally through the superior and inferior orbitary foramina, and next to the outer and inner canthus. After some time, Haley bore about twenty-five pair of plates without much inconvenience.

The quantity of muriatic acid Mr. Whitlam made use of seems larger than what is generally recommended. Mr. Wilkinson, I think, recommends one ounce to a pint. If I mistake not an ingenious lecturer at Sheffield uses only one-thirtieth. The result of my experience is, that with my machine one-twentieth* answers better than if it is either stronger or weaker.

I am, &c.

*Fulneck, near Leeds,*

*Dec.* 29, 1805.

J. WAIBLINGER.

* I mean one ounce of muriatic acid to twenty ounces of water.

